# Condylar Neck and Sub-Condylar Fractures: Surgical Consideration and Evolution of the Technique with Short Follow-Up on Five Cases

**DOI:** 10.3390/dj8040125

**Published:** 2020-11-03

**Authors:** Antonio Cortese, Antonio Borri, Marco Bergaminelli, Fabrizio Bergaminelli, Pier Paolo Claudio

**Affiliations:** 1 Unit of Maxillofacial Surgery, Department of Medicine and Surgery, University of Salerno, 84084 Fisciano (Salerno), Italy; antonio-borri@alice.it (A.B.); marcobergaminelli@gmail.com (M.B.); fbergaminelli@gmail.com (F.B.); 2 Department of BioMolecular Sciences, National Center for Natural Product Research, University of Mississippi, University, MS 38677, USA; 3 Department of Maxillofacial Surgery, University of Mississippi Medical Center, Jackson, MS 39216, USA; 4 Department of Radiation Oncology, University of Mississippi Medical Center, Jackson, MS 39216, USA

**Keywords:** condylar fractures, surgery, surgical access, facial nerve injury

## Abstract

Condylar neck and sub-condylar fractures of the mandible are a frequent occurrence in maxillofacial surgery. The indication for surgical treatment of these fractures has changed over time, and several techniques have been developed with different results in the attempt to avoid the most worrisome adverse event, i.e., facial nerve injury. In this study, we present a new technique that combines an intraoral and a cutaneous pre-auricular access, which allows for easy and safe access to the surgical site, preventing facial nerve injury and avoiding surgical scars in high-impact aesthetic areas of the neck. Five consecutive patients affected by condylar neck or sub-condylar fractures were treated at a single institution using a combined intraoral and pre-auricular access. Results were evaluated after three months from surgery in terms of mandibular mobility, occurrence of complications, and patient’s satisfaction. All five patients had good outcome, with complete healing of the fracture and no occurrence of complications, including no facial nerve palsy. A key point of the technique is the safe reduction of the two mandibular fragments realized by a combined intraoral and a cutaneous pre-auricular surgical access. The periosteal plan of the ramus can be widely and safely elevated with the intraoral approach and connected to the condylar bone plane by the pre-auricular cutaneous approach without any need for soft tissue dissection at the fracture rim, thereby avoiding facial nerve injuries. Wide ramus periosteum elevation creates an “optical space”, allowing fragment reduction and fixation under direct oblique view without any endoscopic need. Our results strongly suggest that with our technique it is possible to treat sub-condylar and condylar neck fractures safely, avoiding facial nerve injury, which is an unacceptable complication due to its heavy impact on a patient’s life.

## 1. Introduction

Condylar fractures of the mandible are a controversial topic in maxillofacial surgery due to the variety of features they manifest, and the wide range of surgical techniques commonly used to treat them. Common disadvantages of surgery for mandibular fractures are scars, salivary gland-associated complications, maxillary artery, or pterygoid plexus injuries, but the greatest concern is facial nerve injury (FNI).

Several surgical approaches have been proposed to treat condylar fractures; however, there is not yet a consensus on the surgical technique to be used to avoid FNI. Minimally displaced fractures can be treated by closed reduction with maxilla–mandibular fixation (CR/MMF), whereas moderately or severely displaced fractures usually need to be treated with an open reduction (OR). Indications for surgery in fractures of the mandibular condyle have changed over time. Zide and Kent described a series of absolute and relative surgical indications that became the gold standard in 1980s (Table 1) [1,2]. However, scientific and technological innovations allowed Zide and Kent’s indications to be overcome. The American Association of Oral and Maxillofacial Surgery (AAOMS) proposed new international guidelines, and we have adopted the AAOMS special committee parameters of care indication for open reduction of condylar fractures (Table 2).

Simply on the basis of anatomical difference for surgical treatment, fractures of the mandibular condyle were commonly distinct in two categories: intra-capsular and extra-capsular fractures. More recently, mandibular condylar fractures were classified on the basis of the site in head (over the polars connecting line), neck (between bipolar and sigmoid noch line), and sub-condylar (under sigmoid noch line), which has also been adopted by the AOCMF for its utility in surgical technique selection [3]. Intra-capsular (head and high neck) fractures are usually approached surgically by a pre-auricular incision (just in front of the tragus). The incision in the pre-auricular area extends to the temporal region. The temporal fascia is incised vertically, and dissection is performed below the temporal fascia until the capsule of the temporo-mandibular joint (TMJ) is approached, preserving the temporal branch of the facial nerve. The TMJ capsule is then incised exposing the fracture. In extra-capsular (low neck and sub-condylar) fractures, percutaneous approaches are mostly used instead, and the mandibular condyle can be reached via deep or superficial routes.

Deep techniques provide submandibular (Risdon) or retro-mandibular approaches whereas superficial techniques are based on trans-parotid or trans-masseter and antero-parotid (TMAP) accesses [3,4].

The submandibular technique starts with an incision following the inferior border of the mandible. The dissection is purely subcutaneous at first, and then the platysma muscle is dissected about 2cm above the mandibular angle, giving access to the masseter fascia. The masseter muscle is then incised in order to reach bone contact. A sub-periosteal dissection is performed upward in direction of the condyle until eventually the fracture area is reached [3,4,5].

The retro-parotid approach is performed through an incision right below the ear lobe towards the mandibular border. The condyle is exposed through a dissection of the subcutaneous tissues posteriorly around the parotid capsule and after retraction of the parotid gland [4]. The trans-parotid approach provides an incision parallel to the posterior border of the mandibular ramus. The fracture is eventually exposed with poor safeguard of the facial nerve by cautiously dissecting the musculo-aponeurotic fascia and the parotid capsule [4,5].

The superficial technique (TMAP) starts with a retro-mandibular incision in the original form, which can be extended to the pre-auricular region in the modified form. Dissection of the superficial muscle-aponeurotic system is performed until reaching the anterior border of the parotid gland, then the parotid is retracted posteriorly, and the condyle is approached passing posteriorly to the masseter muscle. A high cervical TMAP can also be used. In this case, the incision is performed from 0.5 to 1 cm below the inferior mandibular border, and the masseter muscle is reached after a subcutaneous dissection on the platysma muscle and dissection and retraction of the platysma itself. The access to the mandibular ramus is obtained through cutting the muscle anteriorly to the parotid gland in a so-called “nerve-free window” [4]. Recent studies proposed an endoscopic reduction via intraoral approach, which is supposed to reduce the risk of FNI [6,7]. The incision is performed on the gingiva near the 2° or 3° molar, extending posteriorly on the anterior border of the mandibular ramus. The mucosa flap is eventually retracted, exposing the fracture, which can be treated endoscopically with the related difficulties for reduction and fixation of sub-condylar and neck fractures [3,6].

For all these techniques, after exposing the fractures, occlusion is obtained at maximum intercuspation by maxilla–mandibular fixation and the fracture is reduced and fixed mostly by plates. After surgery, maintenance of fixation by elastic bands on skeletal screw anchorages is recommended. In this study, we propose a method that can be used for both intracapsular and extracapsular fractures using a combined cutaneous pre-auricular approach for upper screw positioning and an intraoral transmucosal approach with wide periosteal elevation of the ramous, achieving a large space for transcutaneous lower screw insertion under direct view, preserving the integrity of the facial nerve and avoiding surgical scars in high aesthetic impact areas of the neck.

The main treatment difficulties are related to neck condylar fractures because of the lack of general consensus on the surgical approach that needs to be used in order to avoid the most devastating complication, i.e., facial nerve injury [4,6].

In the recent literature, several studies have been published in the attempt to correlate the incidence of FNI with the different surgical approaches.

Five possible procedures have been described on the basis of the way they cross the division site of the marginalis mandibulae branches of the facial nerve (MMB), following the criteria of classifying surgical access by superficial and deep approaches.

There is also a second classification that divides surgical approaches from most posterior to most anterior, and it has been described that the more posterior the access, the higher is the risk of facial nerve injury [8].

A general increase of side effects from posterior to anterior and from superficial to deep approaches have been described, with the posterior and deep access classified as the riskier for FNI insurgence [3,8]. Another risk factor for FNI is a wide dislocation of fractures, with increased FNI incidence correlated with condyle fracture dislocation [3]. In our opinion, FNI is such a detrimental drawback for condylar neck and sub-condylar fracture treatment, one that is unacceptable due to its heavy consequences in social life because of the importance of face expressivity in human relations. Because of the heavy balance risk/benefit, only safe techniques for condylar fracture treatment should be considered.

In order to limit FNI as little as possible, we proposed a new technique for condylar neck and sub-condylar fracture treatment that has the advantage of limiting the incidence of facial nerve complications also in the presence of dislocated condylar neck and sub-condylar fractures. The new technique is based on the combination of the preauricular approach with an intraoral approach. By using our dual surgical access, risks for FNI may also be limited for dislocated fracture, avoiding wide soft tissue dissections in the division area of the MMB as needed in most common techniques for the reduction and fixation of dislocated condylar neck fragments.

Since the incidence of FNI described in deep approaches is up to 48.1% with submandibular approach and up to 40% with the retro-parotid approach, the superficial approaches are relatively riskless with FNI occurrence of up to 30% in the trans-parotid, 7.7% in TMAP, and 0.6% in high cervical TMAP [4]. The aim of our work was to show the feasibility and the advantages of our technique in comparison to current techniques for condylar fractures in relation to many different factors that are fracture-related, such as site, direction, and characteristics (multiple, comminute) of the fracture, and patient-related, such as age, occlusion, and dentition. We analyzed five cases of condylar fractures we treated using our dual surgical access approach and show here the results obtained, the advantages, and drawbacks of our technique.

## 2. Materials and Methods

### 2.1. Patient Cohort

Five fractured condyles were operated upon since October 2016, with patients’ characteristics being illustrated in Table 3. Five patients with dislocated condylar neck fractures and sub-condylar fractures were included in this study, in accordance with the AAOMS special committee on parameters of care indication for open reduction.

All subjects gave their informed consent for inclusion before they participated in the study. The study was conducted in accordance with the Declaration of Helsinki, and the protocol was approved by the Ethics Committee of University of Salerno (project identification #40, 7 January 2019).

**Figure 1:**
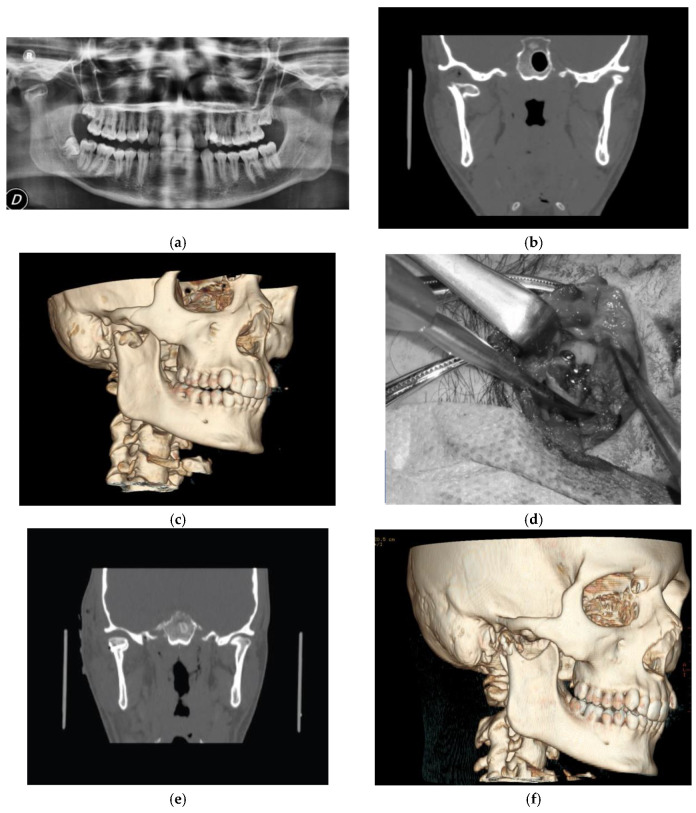
(**a**) Pre-operatory panoramic X-ray; (**b**) pre-operatory computerized tomography scan (CT scan); (**c**) pre-operatory volumetric CT scan; (**d**) intra-operatory view; (**e**) post-operatory coronal CT scan; and (**f**) post-operatory volumetric CT scan of patient #1.

**Figure 2:**
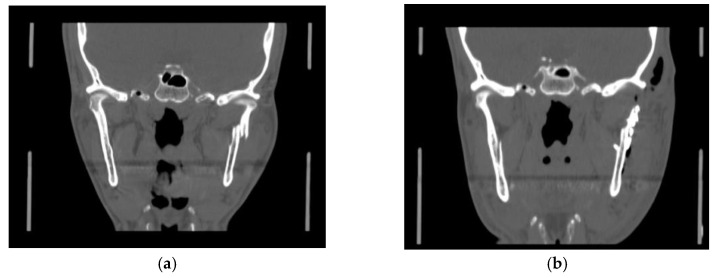
(**a**) Pre- operatory coronal CT scan; (**b**) post-operatory coronal CT scan of patient #2.

**Figure 3:**
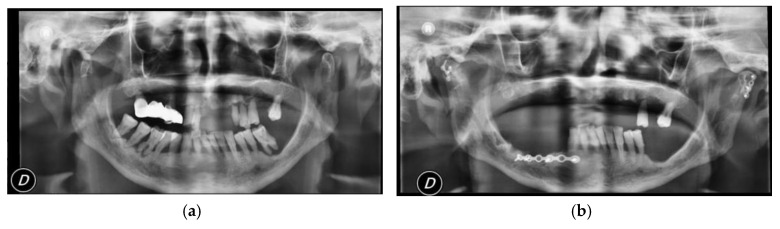
(**a**) Pre-operatory panoramic X-ray; (**b**) post-operatory panoramic X-ray of patient #3.

**Figure 4:**
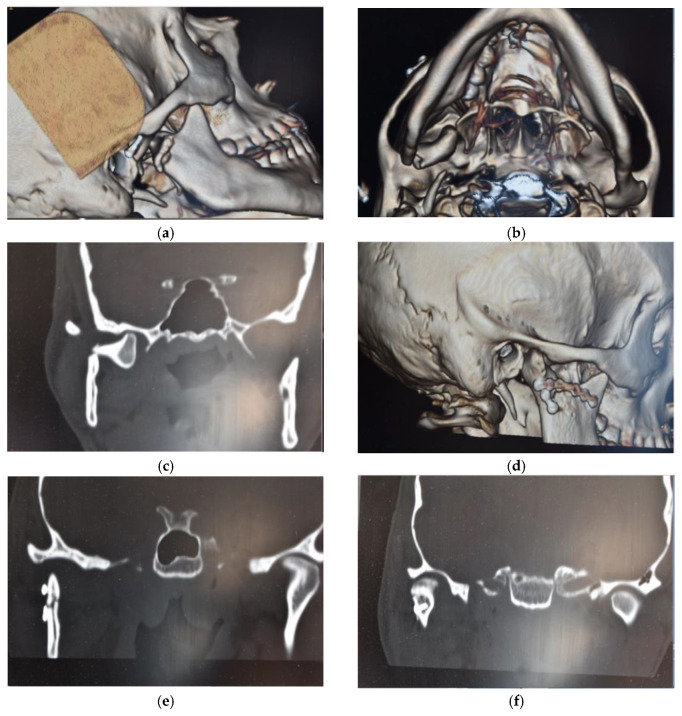
(**a**,**b**) Pre-operatory volumetric CT; (**c**) pre-operatory coronal CT; (**d**) post-operatory volumetric CT; and (**e**,**f**) post-operatory coronal CT of patient #4.

Surgical treatment was performed in patients with widely displaced fractures with lack of contact between the two fragments (Figure 1, Figure 2, Figure 3 and Figure 4), or when the displacement of the fragments caused a lack of function of the TMJ that could not be resolved with conservative treatments, or in multiple fractures of the middle third of the face in order to use the mandible as a guide for replacement of the bones of the middle third of the face, as previously described [8].

A-shaped miniplates and screws (Tekka France) were used in condylar neck fractures, while 2 miniplates were adopted in wide based sub-condylar fractures depending on the fracture rim shape in order to achieve maximum stability in fixation [9].

A-shaped plates are suitable for condylar neck fractures as resulting from finite element analysis (FEA) in the literature [10].

Because use of resorbable plates in maxillofacial fractures is still debated, we selected titanium fixation systems, even if conceptually resorbable plates and screws may represent an advantage particularly in growing patients avoiding permanent limits in district growth and second surgery stage in case of removal necessity [11,12].

### 2.2. Surgical Technique

The rationale of this modified surgical approach for condylar fractures was to obtain the periosteal elevation at the surgical site via a pre-auricular transcutaneous access for the condylar area, combined with a wide periosteal elevation by intraoral approach and ramous preparation for the sub-condylar area, thereby avoiding a submandibular or retro parotid access and in this way reducing the risks for FNI at the fracture rim periosteal elevation time.

The multi steps techniques combining pre-auricular and intraoral approaches followed the surgical flowchart of (1) intraoral incision with periosteal elevation on the mandibular ramous similar to sagittal split surgery; (2) lower temporalis muscle detachment from the coronoid process; (3) pre-auricular incision with periosteal elevation from the lateral side of the condyle; and (4) screw insertion on the condylar head to proper manage the condylar head position without damaging the condylar fragment and avoiding periosteal elevation and pterygoid detachment from the inner side of the condyle, thereby preventing condylar head damaging and necrosis.

Fragment reduction was performed under direct view, combining the pre-auricular and intra-oral access and obtaining a direct view by wide periosteal elevation of the ramous.

Multi-layer closure was performed after fixation with Redon drainage insertion.

**Figure 5:**
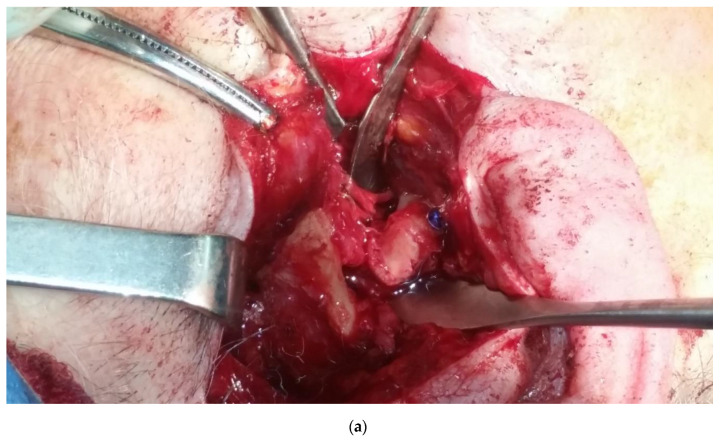

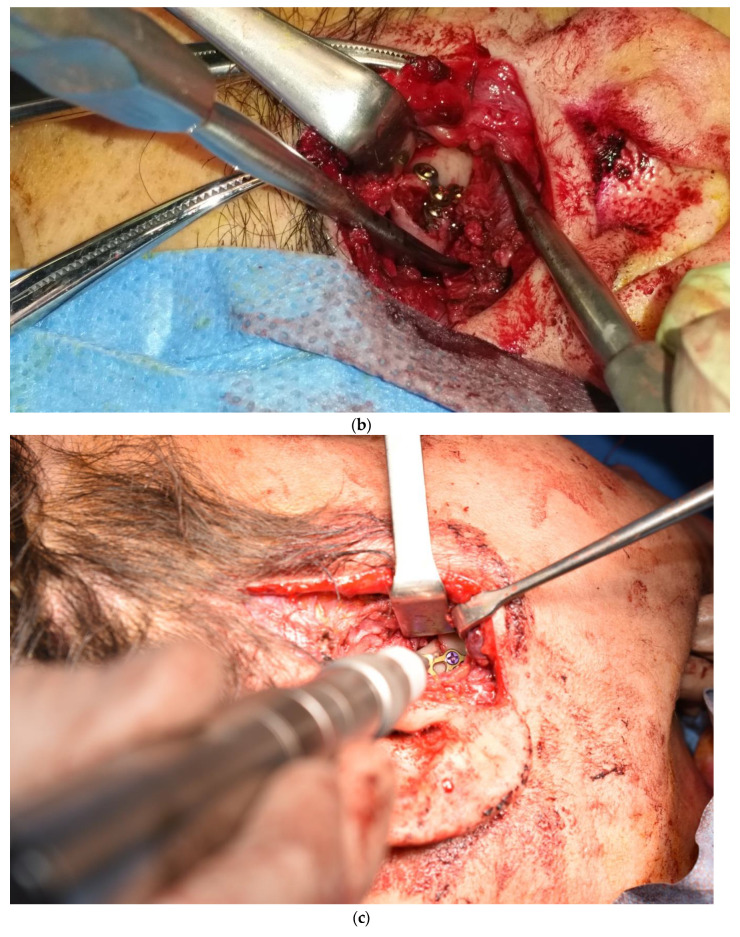
(**a**) Condylar head reduction by screw insertion for safe managing. (**b**) Condylar head fixation. (**c**) Condylar head fixation after lower screws fixation on the ramous.

By this technique, we performed upper screws insertion under direct view of the condylar area by pre-auricular access (Figure 5a), while lower screw positioning on the ramous fragment was placed by cheek trans cutaneous trocar access under direct oblique view from the intraoral mucosa incision. Complete vision of the entire sub-condylar rim fractures was possible from combined pre-auricular and intraoral incisions because of the wide periosteal elevation performed by intraoral mucosa incision approach, after coronoid insertion detachment of the temporalis muscle. In this way, a direct view was created that allowed precise fracture reduction also under finger tactile control for the posterior ramous aspect, avoiding endoscopic technical difficulties (Figure 5c).

In particular, the posterior sub-condylar area was visible by preauricular incision, while the anterior sub-condylar area was also visible through intraoral mucosa access.

Moreover, surgical scars in the aesthetic areas of the neck following current techniques were avoided. Cutaneous incision starts on the upper end of the helix proceeding in the pre-auricular area, extending to the tragus and the surgical path proceeded by deeply reaching the auricular cartilaginous plane and the TMJ capsule. Following opening of the TMJ capsule, we could find the condylar fragment that usually was dragged medially and anteriorly by the insertion of the lateral pterygoid muscle. The surgical path followed the bone plane of the condylar fragment and stopped at the edge of the fracture.

An intraoral incision was performed on the vestibular mucosa at the level of the inferior retromolar trigone running along the anterior border of the ramous, followed by a wide elevation of the entire periosteal plane of the mandibular ramus, including the posterior margin. Following the bone plane by sub-periosteal intraoral route until the fracture edge, we could connect both the preparation of the condylar fragment and the preparation of the mandibular ramus, avoiding FNI risks at the division level of the main facial nerve branches that cross the fracture rim, superficially passing the bone plane.

In detail, once we obtained full control of the fractured area by wide periosteal elevation of the ramous, we could obtain miniplate and screw positioning under direct oblique vision, combining the pre-auricular and the trans-oral approaches by direct trans-cutaneous oblique insertion or via a trans-cutaneous trocar approach. Full control for bone fragment reduction with miniplates and screw positioning could be achieved by a pre-auricular incision for the condylar area (upper screws) and by a transcutaneous access after intraoral periosteal elevation to place sub-condylar lower screws. Even in the case of a sub-condylar fracture limited to the posterior border of the ramous, lower screw positioning for the ramous fragment with proper positioning of the orthogonal axis could be performed by combining a TMAP access, although limiting any parotid or masseter dissection because of the previous wide ramus preparation by intraoral approach, thereby limiting or avoiding FNI risks. In terms of obtaining condylar fragment repositioning and reduction at the fixation time, a digit pressure to the retromolar area of the inferior wisdom teeth or wire traction at the gonion angle by screw insertion is an important maneuver in order to increase the posterior vertical dimension, achieving easier fracture reduction and avoiding dangerous manipulation of the condyle. A long screw partial insertion in the condylar head can be a useful shrewdness for safe traction, positioning, and fixation of the dislocated condyle (Figure 5b).

Additionally, to avoid FNI in the case of full dislocated condylar fractures, after elevation of both the condyle periosteal plane through a pre-auricular access and of the ramous periosteal plane through an intraoral access, we performed the connection of the two planes with a limited dissection at the emerging point of a periosteal elevator inserted from the intraoral access along the bone plane up to the fracture rim. In this way, a very limited dissection of the soft tissues that bridge the two-bone fragment planes at the fracture rim area was performed, avoiding dangerous dissecting maneuvers in the area of the facial nerve main branch division.

### 2.3. Evaluation of Patients’ Results

The results of this dual surgical access technique were evaluated on the basis of dental occlusion, bone fragment alignment after reduction and after fixation, facial nerve functionality, skin scarring, temporomandibular joint functionality, temporomandibular joint symptomatology, and patient satisfaction.

Occlusion functionality was evaluated through checking precise intercuspation and group function guides in protrusive and bilateral jaw movements. Bone fragment alignment was evaluated by panoramic X-ray of the jaws and a computed tomography scan (CT scan) of the facial skeleton on axial and frontal view, with volumetric reconstruction. All the exams were taken pre-operatively and immediately post-op, and an X-ray panoramic alone was taken at 3 months follow-up. Bone fragment reduction and fixation was graded in a scale from 1 to 5, where 1 was worst and 5 was best alignment.

For evaluation of facial nerve function post-operatively, we used the House–Brackmann Facial Nerve Grading System that assesses facial nerve palsy by considering two parameters: the extension of the elevation movements of the middle portion of the eyebrow and the extension of the lateral movements of the angle of the lip. The House–Brackmann system rates 1 point for each 0.25 cm of movement, and the scores are added to reach a maximum of 8 points.

Evaluation of skin scarring was based on the Vancouver Scar System of Baryza, which considers 4 parameters: pliability, vascularity, pigmentation, and scar height. We obtained a rate of the global look of the scar, assigning a score to each parameter and adding the scores up to 13 points. The lower the score, the better the scar will look.

Postoperative TMJ functionality and postoperative TMJ symptomatology were evaluated on the basis of post-operative mandibular motion values, maximum mouth opening, maximum deviation on left side, maximum deviation on right side, and maximum protrusion (Table 4).

Patients’ general satisfaction was evaluated by asking them to grade their satisfaction from 1 to 10 (Table 5). We also recorded the possible occurrence of postoperative complications such as Frey’s syndrome, infection, salivary fistula, and non-union fractures (Table 6).

## 3. Results

All the five cases were evaluated at three months and showed good aesthetic and functional results with proper alignment of the bone fragments. All the five patients showed good mouth opening, with three of five patients opening their mouth more than 40 mm, with measurements taken from the edge of the upper incisor teeth to the edge of the lower incisor teeth (Table 4 and Table 6). Three patients out of five also showed homolateral and contralateral range of movements greater than 8 mm. Two patients out of five showed a maximal protrusion greater than 8 mm (Table 4).

No FNI were detected (Table 6). Only in one case was temporary facial paresis observed after surgery; however, it regressed in two weeks. In this case, facial paresis was probably related to post-operative edema of the soft tissues at the surgical site. No patient experienced facial pain post-surgery, and surgical wounds showed good healing with an average score of 1 on the basis of the Vancouver Scar System of Baryza.

## 4. Discussion

Surgical indications for condylar neck and sub-condylar fractures are widely debated in the literature because of the significant risk of FNI complications during surgery. No general consensus has been reached on the selection of the surgical technique to be used to avoid FNI, and FNI incidence is still the most important complication, even after selecting the most recently developed surgical techniques.

A review of the literature using keywords for surgical technique selection and complication incidences related to FNI following condylar neck and sub-condylar fractures, as well as in relation to the plane of dissection, mainly showed five techniques. In relation to the surgical approach and incidence of FNI, surgical techniques can be divided into deep and superficial, posterior and anterior, depending on the site of dissection and the amount of dislocation, with increased FNI incidence being correlated to increased dislocation [13,14].

The main reason for FNI after condylar fracture treatment is related to the marginalis mandibulae branch damage of the VII cranial nerve because of its terminal pattern of innervation and division site, which runs close to the surgical field.

A possible explanation of these negative events can also be correlated to the amount of soft tissue traction and dissection during surgery (the more traction as is the case in deep techniques, the higher the FNI risks), as well as the site and amount of dissection (lower risks in anterior dissections for plexiform pattern of the facial nerve at anterior sites with free risk area for FNI at ante-parotid site), in comparison to posterior dissections.

Regarding the higher FNI risk observed in condylar fractures with dislocated fragments, the correlation can be explained by the necessity of soft tissue dissection at the fracture site, where the main branches of the facial nerve run across the fracture rim. Using our technique, the dissection of soft tissue can be limited by a periosteal elevator insertion and use as a guide at the condylar and ramous fragments fracture rims.

After both a pre-auricular incision and a mucosa intraoral access, both fields were connected by periosteal elevation running along the ramous and the condyle bone planes, without any need for further soft tissue dissection at the condylar neck fracture site.

A key point of the technique we described is the safe connection of the sub-periosteal planes of the two mandibular fragments: condyle and ramus. Connection of the two periosteal elevation planes at the condyle area by pre-auricular approach and at the ramus fragment by intraoral approach after wide ramus periosteal elevation could be safely achieved without any wide soft tissue dissection in the sub-condylar area through simply connecting the bone surfaces of the two mandibular fragments by a periosteum elevator. In this way, selective and limited soft tissue dissection under elevator tip guide was possible in the sub-condylar area to prepare ramous stump, limiting dangerous maneuvers for main branch integrity of the facial nerve.

Fracture reduction and fixation can be achieved under direct oblique view with the aid of a head light at the condyle level by the pre-auricular access for upper screw insertion on the condylar upper fragment and by intraoral access after the mentioned wide periosteal elevation by trans-oral mucosa view for lower screw insertion by trocar trans-cutaneous cheek access. Following our technique, lower screw area view could be achieved by preauricular trans-cutaneous (sub-condylar rims) or intraoral (condylar neck) access after wide ramous periosteal elevation, creating an optical view space. Moreover, in this way, reduction and precise alignment can be checked by finger tactile control through the oral access, avoiding endoscopic difficulties.

In our opinion, periosteal plane elevation for ramus fragment identification by pre-auricular, submandibular, or retro-parotid cutaneous approaches are dangerous for FNI because of the soft tissue dissection in the division area of the facial nerve branches when connecting the two different sub-periosteum dissection planes of the two dislocated mandibular fragments at reduction and fixation time.

The condylar fragment is commonly dislocated anteriorly and medially because of the external pterygoid muscle traction, while the mandibular ramus fragment is normally located on a more superficial plane in condylar neck fractures [8]. Approaching these fractures by a pre-auricular cutaneous incision alone is dangerous for FNI because the dissection must proceed from the cutaneous plane to the periosteal plane of the mandibular ramus, passing through the subcutaneous and muscular planes that contain vessels and branches of the VII cranial nerve with associated injury risks. Moreover, the submandibular or retro-mandibular approaches are dangerous for FNI, with increased risks for deep and posterior approaches due to the soft tissue dissection closer to the mandibular branch of the facial nerve.

Adopting our technique, the periosteal plane of the mandibular ramus can be widely and safely elevated by the intraoral approach, thus allowing a good view of the sub-condylar rim by the combination of the pre-auricular access with the trans-oral access, avoiding the need for an endoscopic device with related difficulties. Lower screws in the posterior ramus area can be inserted by the pre-auricular approach, while lower screws in the anterior sub-condylar area can be inserted through a cheek trans-cutaneous access by trocar under intraoral vision after wide temporalis muscle insertion detachment on the coronoid process, avoiding any risk for FNI, which is the main complication in condylar neck and sub condylar fractures.

Even in the case of difficulties in fixing sub-condylar fractures at the posterior mandibular border by our technique, the combination with a trans-parotid or trans-masseter and antero-parotid (TMAP) approach can be performed, but through reducing the extent of soft tissue dissection because of the wide periosteal elevation already performed at the ramus area by the intraoral approach with less risks for FNI.

A possible evolution of the technique will consist of digital planning of the operation through also using custom made fixation plates, reducing in this way the incidence of fragment malposition [15].

## 5. Conclusions

FNI is a detrimental drawback for condylar neck and sub-condylar fracture treatment, which is unacceptable due to its heavy consequences on social life because of the importance of face expressivity in human relations. Because of the risk/benefit balance, only safe techniques for condylar fractures treatment should be considered.

Our results suggest that a combined pre-auricular and intraoral approach for surgical treatment of sub-condylar and condylar neck fractures can be used to avoid facial nerve injuries.

The main advantages of the technique we described are

(1) Increased safety for FNI.
(2) Less scarring, with the possibility to make a cutaneous incision only in the pre-auricular area where scars are almost invisible and aesthetically well tolerated.
(3) Precision in final results with the ability to reduce and fix the fractured mandibular fragments under direct view by combination of multiple approaches.
(4) Expansion in indication for open surgery in neck and sub-condylar fractures because of improved functional and aesthetic results, while reducing risks of complications.

The main disadvantage is the complexity of the technique, with related time extension needed at surgery.

## Figures and Tables

**Table 1:** Zide and Kent’s indications for open reduction (1983) [1,2].

**Absolute Indications**
Displacement into middle cranial fossa
Impossibility of obtaining adequate occlusion by closed reduction
Lateral extracapsular displacement
Invasion by foreign body
**Relative Indications**
Bilateral condylar fractures in an edentulous patient without a splint
Unilateral or bilateral condylar fractures with a comminute midfacial fracture, prognathism, or retroprognathism
Periodontal problems
Loss of teeth
Unilateral condylar fracture with unstable base

**Table 2:** The American Association of Oral and Maxillofacial Surgery (AAOMS) special committee on parameters of care indications for open reduction [3].

Physical evidence of fracture
Imaging evidence of fracture
Malocclusion
Mandibular dysfunction
Abnormal relationship of jaw
Presence of foreign bodies
Lacerations and/or hemorrhage in external auditory canal
Hemotympanum
Cerebrospinal fluid otorrhea
Effusion
Hemarthrosis

**Table 3:** Gender, age, and type of trauma of neck condylar fractures or sub-condylar neck fractures.

Patient	Gender	Age	Cause of Trauma	Type of Fracture
**1**	M	28	Sports trauma	Dislocated condylar neck, right
**2**	M	53	Car accident	Dislocated sub-condylar, left
**3**	F	81	Domestic accident	Dislocated condylar neck, bilateral
**4**	M	26	Fall	Dislocated sub-condylar, right
**5**	F	45	Car accident	Dislocated sub-condylar, left

**Table 4:** Post-operative mandibular motion values in the unilateral condylar fractures. Results are three months post-treatment.

Physiological Values of the Mandibular Motion	Number of Patients
**Maximum Mouth Opening (mm)**	
>40 mm	3
<40 mm	2
**Lateral Movement, Contralateral to the Side of the Fracture (mm)**	
>8 mm	3
<8 mm	2
**Lateral Movement, Homolateral to the Side of the Fracture (mm)**	
>8 mm	3
<8 mm	2
**Maximal Protrusion (mm)**	
>8 mm	2
<8 mm	3

**Table 5:** Patients’ satisfaction. Results at three months after treatment.

Patient Number	Satisfaction Level
**1**	9
**2**	9
**3**	8
**4**	10
**5**	9

**Table 6:** Post-operative results and complications.

Patient	Mouth Opening at 1 Month (in mm)	Mouth Opening Pattern (Deviation in mm)	Facial Pain	Post-Operative Complications *	Surgical Scarring
**1**	38	0	No	0	1
**2**	42	0	No	0	1
**3**	40	1	No	0	2
**4**	35	1	No	0	0
**5**	41	2	No	0	1

* Frey’s syndrome, fracture of the plate, infection, salivary fistula, permanent paralysis of facial nerve.
